# Prognostic role of metabolic parameters of ^18^F-FDG PET-CT scan performed during radiation therapy in locally advanced head and neck squamous cell carcinoma

**DOI:** 10.1007/s00259-015-3104-8

**Published:** 2015-06-17

**Authors:** Myo Min, Peter Lin, Mark T. Lee, Ivan Ho Shon, Michael Lin, Dion Forstner, Victoria Bray, Andrew Chicco, Minh Thi Tieu, Allan Fowler

**Affiliations:** Cancer Therapy Centre, Liverpool Hospital, Liverpool, NSW 2170 Australia; University of New South Wales, Sydney, NSW Australia; Ingham Institute of Applied Medical Research, Liverpool, NSW Australia; Department of Nuclear Medicine and Positron Emission Tomography, Liverpool Hospital, Liverpool, NSW Australia; Department of Radiation Oncology, Calvary Mater Newcastle, Newcastle, NSW 2298 Australia; University of Western Sydney, Sydney, NSW Australia; University of Newcastle, Newcastle, NSW Australia

**Keywords:** Head and neck cancer, Prognostic value, FDG PET CT, Adaptive radiotherapy, Metabolic parameters

## Abstract

**Purpose:**

To evaluate the prognostic value of ^18^F-FDG PET-CT performed in the third week (iPET) of definitive radiation therapy (RT) in patients with newly diagnosed locally advanced mucosal primary head and neck squamous-cell-carcinoma (MPHNSCC).

**Methodology:**

Seventy-two patients with MPHNSCC treated with radical RT underwent staging PET-CT and iPET. The maximum standardised uptake value (SUV_max_), metabolic tumour volume (MTV) and total lesional glycolysis (TLG) of primary tumour (PT) and index node (IN) [defined as lymph node(s) with highest TLG] were analysed, and results were correlated with loco-regional recurrence-free survival (LRFS), disease-free survival (DFS), metastatic failure-free survival(MFFS) and overall survival (OS), using Kaplan-Meier analysis.

**Results:**

Optimal cutoffs (OC) were derived from receiver operating characteristic curves: SUV_max-PT_ = 4.25 g/mL, MTV_PT_ = 3.3 cm^3^, TLG_PT_ = 9.4 g, for PT, and SUV_max-IN_ = 4.05 g/mL, MTV_IN_ = 1.85 cm^3^ and TLG_IN_ = 7.95 g for IN. Low metabolic values in iPET for PT below OC were associated with statistically significant better LRFS and DFS. TLG was the best predictor of outcome with 2-year LRFS of 92.7 % vs. 71.1 % [*p* = 0.005, compared with SUV_max_ (*p* = 0.03) and MTV (*p* = 0.022)], DFS of 85.9 % vs. 60.8 % [*p* = 0.005, compared with SUV_max_ (*p* = 0.025) and MTV (*p* = 0.018)], MFFS of 85.9 % vs. 83.7 % [*p* = 0.488, compared with SUV_max_ (*p* = 0.52) and MTV (*p* = 0.436)], and OS of 81.1 % vs. 75.0 % [*p* = 0.279, compared with SUV_max_ (*p* = 0.345) and MTV (*p* = 0.512)]. There were no significant associations between the percentage reduction of primary tumour metabolic parameters and outcomes. In patients with nodal disease, metabolic parameters below OC (for both PT and IN) were significantly associated with all oncological outcomes, while TLG was again the best predictor: LRFS of 84.0 % vs. 55.3 % (*p* = 0.017), DFS of 79.4 % vs. 38.6 % (*p* = 0.001), MFFS 86.4 % vs. 68.2 % (*p* = 0.034) and OS 80.4 % vs. 55.7 % (*p* = 0.045).

**Conclusion:**

The metabolic parameters of iPET can be useful predictors of patient outcome and potentially have a role in adaptive therapy for MPHNSCC. Among the three parameters, TLG was found to be the best prognostic indicator of oncological outcomes.

## Introduction

The loco-regional control of mucosal primary head and neck squamous cell carcinoma (MPHNSCC) has improved significantly using primary radiation therapy (RT) and concurrent systemic therapy [1]. However, these treatments are associated with significant long-term toxicity, which can have a lasting impact on quality of life [2]. Frequently recurrences occur within the initial gross tumour volume [3], and strategies that can better identify tumour radioresistance and selection of appropriate treatment intensification may improve outcomes.

2-[18F] fluoro-2-deoxy-D-glucose positron emission tomography-computed tomography (FDG PET-CT) has an established role in assessment of treatment outcome after completion of organ-preserving chemo-RT for locally advanced MPHNSCC. This is usually performed at 3 to 4 months after completion of treatment with high negative predictive value and low to moderate positive predictive value for tumour recurrence [4]. Obtaining FDG PET-CT at an earlier time point can result in poorer specificity due to treatment-related inflammation. Research evaluating the role of FDG PET-CT in assessing early treatment response during RT is limited to small studies with mixed results [5–8].

The maximum standardised uptake value (SUV_max_) has been the most commonly used PET metabolic parameter for staging and monitoring of treatment response. More novel parameters being assessed include the metabolic tumour volume (MTV) and total lesional glycolysis (TLG). These measurements provide volumetric information on glucose metabolism of the tumour. A recent meta-analysis reported that MTV and TLG from staging FDG PET-CT are additional and independent prognostic indicators for patients with MPHNSCC [9]. There is no published data evaluating the role of MTV or TLG during RT for MPHNSCC. Furthermore, there is no study to date that has reported the prognostic value of the nodal disease during RT in MPHNSCC.

This study aims to investigate the utility of FDG PET-CT performed in the third week of primary RT (iPET) as a prognostic indicator for MPHNSCC. Specifically, we aimed to assess whether the residual metabolic tumour burden measured by SUV_max_, MTV and/or TLG correlated with patient outcomes. The secondary aim was to assess whether the metabolic response, as assessed by percentage reduction of these three metabolic parameters, can also predict treatment outcome.

## Materials and methods

### Study population

Patients with biopsy-proven, newly diagnosed locally advanced MPHNSCC treated by primary RT with curative intent were retrospectively reviewed as part of a trial approved by the local research ethics committee (Sydney South West Area Health Service Human Research Ethics Committee). Only patients with both staging and mid treatment FDG PET-CT performed during the third week of RT were included for analysis. Patients with early stage diseases, nasopharyngeal cancer (NPC) or being treated with RT only were excluded.

### Imaging technique

The studies were acquired in RT treatment position on either a Philips Gemini-GXL-PET-CT (*n* = 48) or a GE Discovery-710 PET-CT (*n* = 24). Patients received between 4.1 (for GE) and 5.18 (for Philips) MBq/kg of FDG after at least 4 hours of fasting. The average blood sugar level was 5.7 ± 1.2 mmol/L (range: 3.3-9.6 mmol/L). The staging and all sequential post-treatment scans were performed on the same scanner with the same acquisition and reconstruction protocols.

The PET studies were acquired in three-dimensional (3D) mode for a total acquisition time of 1.5 – 2.5 min per bed position adjusted according to the patient weight, from vertex to proximal femora at about 1-hour post injection. Transmission scans and attenuation corrections were obtained by using CT: a Philips Brilliance 6-slice CT or a 64-slice GE CT, using helical mode without the use of a contrast medium. CT images were acquired at 3.75 to 5 mm slice thickness and reconstructed to a transaxial matrix size of 512 × 512. The current (30–40 mAs) and voltage (120–140 kV) were varied according to the patient weight. The PET images were reconstructed using a Philips Line of Response-Row Action Maximum Likelihood Algorithm (LOR-RAMLA) or GE VUE Point FX (Time of Flight) algorithm into a 144 × 144 (for Philips) or 256 × 256 (for GE) matrix size with a slice thickness of 3.75 to 4.0 mm.

### FDG PET image interpretation and metabolic parameter measurement

All FDG-PET images were analysed by consensus reading by two nuclear medicine physicians and a radiation oncologist, blinded to clinical data except the primary tumour site. The semi-quantitative analysis was performed on an Advantage Workstation (GE Healthcare) using the PET-VCAR (Volume Computer-Assisted Reading) software (version 1.0). The maximum SUV (SUV_max_) was derived by selecting the most intensely avid area of uptake at the primary tumour on the axial slice. The SUV value was derived as follows: SUV = $\frac{C\left( Bq/ ml\right)}{\left(\frac{A(Bq)}{m(g)}\right)}$ (decay-corrected administered activity [KBq] per millilitre of tissue volume)/(injected FDG activity [KBq]/body weight in gram). The MTV was derived by applying a fixed SUV threshold of 2.5 as the lowest limit of the segmentation criteria. The computer-assisted, automatically derived contouring margins and regions of interest (ROIs) for both measurements were checked on three sectional images (axial, coronal and sagittal) to ensure accurate inclusion of primary tumour and nodal sites, and exclusion of adjacent normal structures. The single-component modality was deselected to prevent the segmentation that may potentially derive from grabbing normal structures outside the region of interest (ROI). The TLG was calculated according to the formula: TLG = mean SUV x MTV. The fixed SUV threshold of 2.5 was chosen because it was recommended as an appropriate criterion in a recent meta-analysis, and is commonly used and associated with prognostic outcome [9]. SUVmax, MTV and TLG were derived for both primary tumour (PT) and index nodes (IN), which is defined as a lymph node or confluent nodal group with the highest TLG reflecting highest metabolic burden.

### Treatment

All patients were treated with IMRT or helical TomoTherapy®: total treatment dose to the GTV was 60-70Gy (2–2.2Gy/fraction); high risk cervical lymph node regions received 60-66Gy (1.8-2Gy/fraction), and the low risk regions received 56Gy (1.6-1.7Gy/fraction). All patients were treated with systemic therapy (chemotherapy or Cetuximab). Management of all cases were reviewed and consensus reached in our Head and Neck multidisciplinary team meetings (HNMDT) prior to commencing treatment.

### Statistical analysis

The predictive accuracy of all three metabolic parameters (absolute values and percentage reductions) of primary tumour (PT) and index nodes (IN) for treatment outcomes was evaluated using receiver operating characteristic (ROC) analysis with the area under the curve (AUC) as an index of accuracy. Optimal cutoffs (OC) for analysis were derived from the ROC curves aiming for best sensitivity and specificity. Time to local, regional or distant failures and survival times were calculated from the date of staging FDG PET-CT. Disease-free survival (DFS), loco-regional failure-free survival (LRFS), metastatic failure-free survival (MFFS) and overall survival (OS) curves were estimated using Kaplan-Meier (KM) analysis and compared using the log-rank (Mantel-Cox) test.

Cox proportional hazards models with 95 % confidence interval and multivariate analysis were performed using clinical confounders (smoking, alcohol consumption, T stage, N stage and Overall AJCC stages). The Pearson correlation test (two-tailed) was used to evaluate the correlation between SUV_max_, MTV and TLG. Statistical significance was considered when the p value was ≤ 0.05 and all levels of significance were two sided. Statistical analysis was performed using IBM SPSS Statistics, version 22.0.

## Results

### Study population

Seventy-two consecutive patients from January 2009 to September 2014 were included in this analysis. Primary tumour sites were oropharynx (*n* = 47), larynx (*n* = 16), hypopharynx (*n* = 6) and oral cavity (*n* = 3). Staging based on AJCC 7th Edition included 18 in stage III and 54 in IV. Patient characteristics and treatment details are summarised in Table 1. The median metabolic values of PT for staging PET vs. iPET were: SUV_max_: 10.9 (range 3.6-27.0) vs. 4.85 (1.1-17.2) g/mL; MTV:17.6 (1.8-77.3) vs. 4.0 (0–48) cm^3^; and TLG: 89.1 (5.1-629.5) vs. 14.15 (0–298.1) g. The median metabolic values of index node for staging PET vs. iPET were: SUV_max_:7.5 (range 1.3-27) vs. 4.0 (0–11.6) g/mL; MTV:10.5 (<0.1-248.8) vs. 2.2 (0–37.2) cm^3^; and TLG:45.6 (<0.1-2120.7) vs. 7.2 (0–169.9) g.

**Table 1:** Patient/tumour characteristics and treatment summary

Total		72
Follow-up	Median	25 months
	Range	6–70 months
Age	Median	60
	Range	(39–75)
Sex	Male	61 (84.7 %)
	Female	11 (15.3 %)
Smoking history	> 10 pack/year	41 (56.9 %)
	≤ 10 pack/year	7 (9.7 %)
	Pack/year not known	11 (15.3 %)
	Non-smoker	13 (18.1 %)
Alcohol History	Heavy (> 3 standard drinks)	22 (30.6 %)
	Non-heavy (≤ 3 standard drinks)	34 (47.2 %)
	Non drinker	11 (15.3 %)
	No record	5 (6.9 %)
Primary tumour site	Oropharynx	47 (65.3 %)
	Larynx	16 (22.2 %)
	Hypopharynx	6 (8.3 %)
	Oral cavity	3 (4.2 %)
T stage	1	6 (8.3 %)
	2	25 (34.7 %)
	3	31 (43.1 %)
	4	10 (13.9 %)
N stage	0	9 (12.5 %)
	1	11 (15.3 %)
	2	47 (65.3 %)
	3	5 (6.9 %)
Staging (Overall)	III	18 (25.0 %)
	IV	54 (75.0 %)
Treatment	Radiotherapy + chemotherapy	57 (79.2 %)
	Chemoradiotherapy (Weekly Cisplatin)	42 (58.3 %)
	Chemoradiotherapy (Weekly Carboplatin)	12 (16.7 %)
	Chemoradiotherapy (3 weekly Cisplatin; Carboplatin + 5-Fluorouracil or Carboplatin + Vinorelbine	3 (4.2 %)
	Induction chemotherapy	17 (23.6 %)
	Radiotherapy + Cetuximab	15 (20.8 %)

At the time of analysis, 56 patients (78 %) were alive and 51 (71 %) were disease free, while 21 (29 %) had treatment failure (15 with loco-regional failure, 11 with distant failure, and five with both loco-regional and distant failure). The median follow-up (of both alive and dead patients) was 25 months (range 6–70, mean 29 months) while that of the surviving patients was 30 months (range 6–70, mean 32 months).

### Correlation of residual absolute metabolic value in primary tumour and treatment outcome

**Fig. 1:**
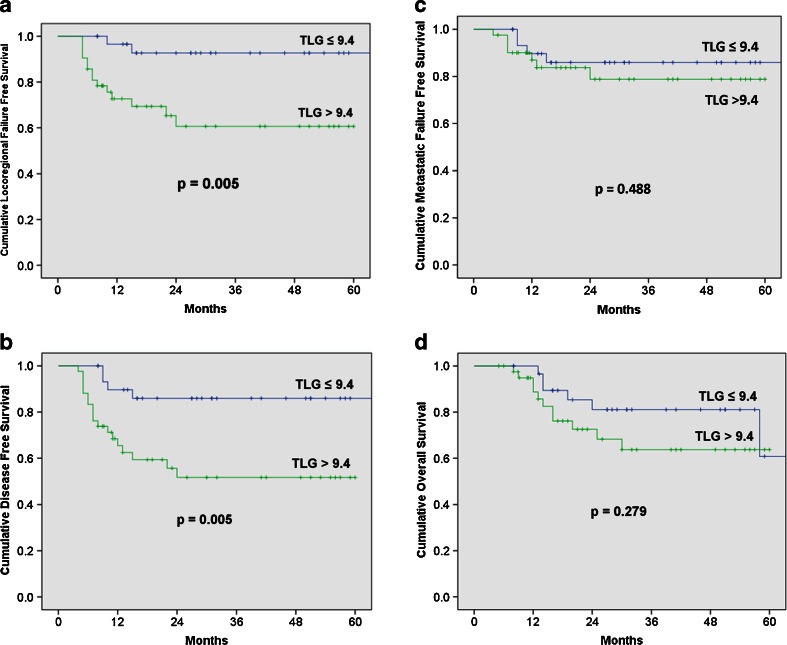
Oncological outcomes according to the total lesional glycolysis (TLG) of the primary tumour during radiation therapy (RT). (**a**) Loco-regional failure-free survival; (**b**) Disease-free survival; (**c**) Metastasis-free survival; (**d**) Overall survival

Optimal cutoffs (OC) of PT for predicting treatment outcomes derived from the ROC curves were: SUV_max-PT_ = 4.25 g/mL, MTV_PT_ = 3.3 cm^3^ and TLG_PT_ = 9.4 g. Low metabolic values of PT in iPET below OC were associated with statistically significant better LRFS and DFS but not OS. TLG was the best predictor of outcome with 2-year LRFS of 92.7 % vs. 71.1 % [*p* = 0.005, compared with SUV_max_ (*p* = 0.03) and MTV (*p* = 0.022)], DFS of 85.9 % vs. 60.8 % [*p* = 0.005, compared with SUV_max_ (*p* = 0.025) and MTV (*p* = 0.018)], MFFS of 85.9 % vs. 83.7 % [*p* = 0.488, compared with SUV_max_ (*p* = 0.52) and MTV (*p* = 0.436)], and OS of 81.1 % vs. 75.0 % [*p* = 0.279, compared with SUV_max_ (*p* = 0.345) and MTV (*p* = 0.512)]. Figure 1 demonstrates the KM survival analysis results of TLG_PT_ based on optimum cutoff values.

Associations between metabolic parameters and treatment outcomes from both univariate and multivariate analyses using Cox Proportional Hazards Model are shown in Table 2. TLG was the only parameter with significantly improved association with oncological outcomes (DFS and LRFS) in both univariate and multivariate analyses.

**Table 2:** Correlation of metabolic values of primary tumour below optimal cutoffs on ROC with treatment outcomes: all patients

	Value	Number		2-year Kaplan-Meier	p value	Cox regression analysis (univariate analysis)			Cox regression analysis, adjusted for confounders^*^ (multivariate analysis)		
						HR	95 % CI	p value	HR	95 % CI	p value
SUV_max_	≤ 4.25	30	LRFS	88.80 %							
	> 4.25	42		74.90 %	0.03	3.666	1.031–13.031	0.045	3.875	0.878–17.105	0.074
	≤ 4.25	30	DFS	82.20 %							
	> 4.25	42		64.50 %	0.025	2.975	1.087–8.143	0.034	4.182	1.15–15.208	0.03
	≤ 4.25	30	MFFS	85.90 %							
	> 4.25	42		84.00 %	0.52	1.490	0.435–5.1	0.525	1.353	0.281–6.523	0.706
	≤ 4.25	30	OS	80.80 %							
	> 4.25	42		75.00 %	0.345	1.616	0.587–4.452	0.353	2.549	0.745–8.721	0.136
MTV	≤ 3.3	32	LRFS	89.80 %							
	> 3.3	40		68.50 %	0.022	3.913	1.101–13.911	0.035	4.725	0.958–23.316	0.057
	≤ 3.3	32	DFS	83.20 %							
	> 3.3	40		62.30 %	0.018	3.143	1.149–8.598	0.026	4.929	1.288–18.858	0.02
	≤ 3.3	32	MFFS	86.30 %							
	> 3.3	40		78.40 %	0.436	1.618	0.473–5.536	0.443	1.502	0.311–7.264	0.613
	≤ 3.3	32	OS	78.30 %							
	> 3.3	40		73.00 %	0.512	1.389	0.515–3.745	0.516	1.868	0.531–6.571	0.33
TLG	≤ 9.4	31	LRFS	92.70 %							
	> 9.4	41		71.10 %	0.005	6.312	1.42–28.057	0.015	8.305	1.485–46.451 %	0.016
	≤ 9.4	31	DFS	85.90 %							
	> 9.4	41		60.80 %	0.005	4.14	1.39–12.332	0.011	7.756	1.847–32.572	0.005
	≤ 9.4	31	MFFS	85.90 %							
	> 9.4	41		83.70 %	0.488	1.537	0.449–5.260	0.494	1.502	0.311–7.264	0.613
	≤ 9.4	31	OS	81.10 %							
	> 9.4	41		75.00 %	0.279	1.736	0.628–4.799	0.288	2.488	0.71–8.72	0.154

SUV_max_ = maximum standardised uptake value; MTV = metabolic tumour volume; TLG = total lesional glycolysis; DFS = disease-free survival; LRFS = loco-regional failure-free survival; MFFS = metastatic failure-free survival; OS = overall survival

^*^confounders: smoking status, alcohol status, T stage, N stage, Overall stage

**Fig. 2:**
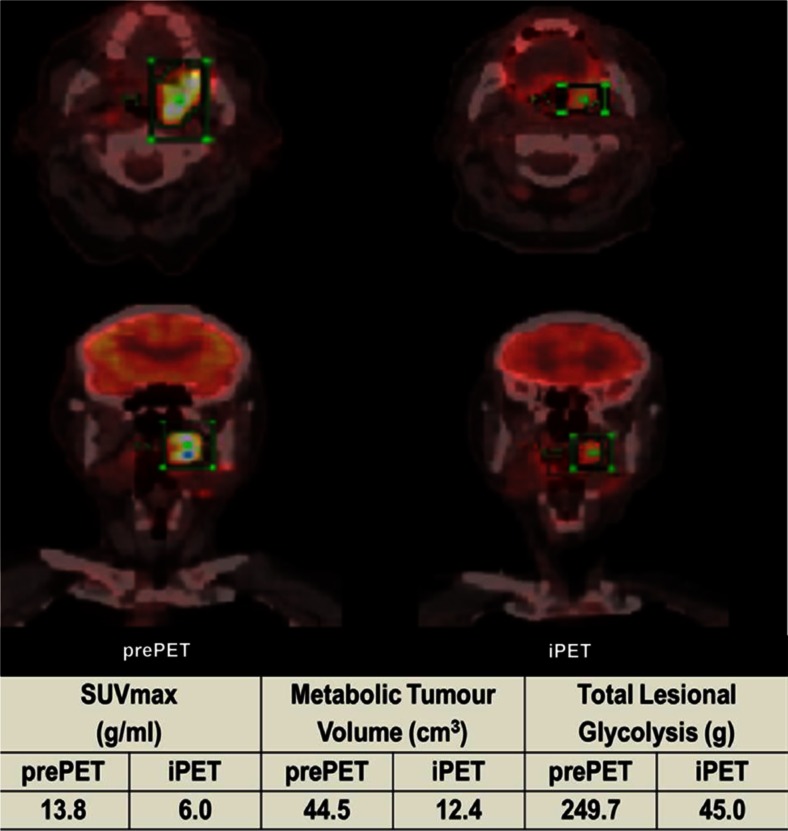
A patient with a good metabolic response (>50 % reduction in all metabolic parameters), but iPET metabolic values above the optimal cutoffs. pre-PET = pre-treatment PET; iPET = mid-treatment (week 3) PET

Pearson correlation tests showed highly significant correlation between all metabolic values of PT where the significance was higher between MTV and TLG: 0.837, 0.838 and 0.976 for SUV_max_ vs. MTV, SUV_max_ vs. TLG and MTV vs. TLG, respectively. Figure 2 shows an example of a patient (oropharyngeal cancer) with a good metabolic response (> 50 % decrease in all metabolic parameters); however, the patient had SUV_max_/MTV/TLG values above OC, and subsequently developed loco-regional failure at 3 months after completion of chemoradiotherapy.

### Percentage reduction in metabolic values in primary tumour and treatment outcome

Because of poor accuracy assessed by ROC analysis, the median values were used as the optimal cutoffs: SUV_max_ = 54.5 %; MTV = 66.9 %; and TLG = 78.3 %. There were no significant associations between the percentage reduction of any metabolic parameters and oncological outcomes.

### Subgroup analysis in concurrent chemotherapy group without Cetuximab

Subgroup analyses were also performed to evaluate the correlation of metabolic parameters and outcomes in patients treated with RT and concurrent chemotherapy, excluding Cetuximab. TLG below the OC in iPET remains the best and only metabolic parameter associated with improved DFS and LRFS, and a trend towards improved OS (Table 3). Both SUV_max_ and MTV did not show any significant correlation with disease outcomes on subgroup analysis. Table 3 shows a comparison between two groups: all patients versus those treated with chemo-RT (excluding Cetuximab).

**Table 3:** Correlation of metabolic values of primary tumour below optimal cutoffs with treatment outcomes: patients treated with concurrent radiation therapy and systemic therapy (with and without Cetuximab)

	Radiation Therapy with concurrent systemic therapy including Cetuximab (*n* = 72)					Radiation Therapy with concurrent systemic therapy excluding Cetuximab (*n* = 57)				
	Value	Number		2 year Kaplan-Meier	p value	Value	Number		2-year Kaplan-Meier	p value
SUV_max_	≤ 4.25	30	DFS	82.20 %		≤ 4.25	24	**DFS**	81.70 %	
	> 4.25	42		64.50 %	**0.025**	> 4.25	33		57.70 %	0.064
	≤ 4.25	30	LRFS	88.80 %		≤ 4.25	24	**LRFS**	85.60 %	
	> 4.25	42		74.90 %	**0.03**	> 4.25	33		67.00 %	0.126
	≤ 4.25	30	OS	80.80 %		≤ 4.25	24	**OS**	81.10 %	
	> 4.25	42		75.00 %	0.345	> 4.25	33		66.50 %	0.308
MTV	≤ 3.3	32	DFS	83.20 %		≤ 3.3	25	**DFS**	82.40 %	
	> 3.3	40		62.30 %	**0.018**	> 3.3	32		56.00 %	0.072
	≤ 3.3	32	LRFS	89.80 %		≤ 3.3	25	**LRFS**	86.50 %	
	> 3.3	40		68.50 %	**0.022**	> 3.3	32		65.20 %	0.132
	≤ 3.3	32	OS	78.30 %		≤ 3.3	25	**OS**	76.50 %	
	> 3.3	40		73.00 %	0.512	> 3.3	32		69.90 %	0.68
TLG	≤ 9.4	31	DFS	85.90 %		≤ 9.4	24	**DFS**	85.90 %	
	> 9.4	41		60.80 %	**0.005**	> 9.4	33		54.30 %	**0.022**
	≤ 9.4	31	LRFS	92.70 %		≤ 9.4	24	**LRFS**	90.20 %	
	> 9.4	41		71.10 %	**0.005**	> 9.4	33		63.20 %	**0.038**
	≤ 9.4	31	OS	81.10 %		≤ 9.4	24	**OS**	80.20 %	
	> 9.4	41		75.00 %	0.279	> 9.4	33		64.60 %	0.37

SUV_max_ = maximum standardised uptake value; MTV = metabolic tumour volume; TLG = total lesional glycolysis; DFS = disease-free survival; LRFS = loco-regional failure-free survival; OS = overall survival

### Correlation of metabolic values in the index node and treatment outcome

Sixty-three patients had node positive disease, but one of them had an excisional biopsy of the neck node prior to the commencement of RT, and therefore, nodal FDG-PET data was obtained from 62 patients. Optimal cutoffs of IN for predicting treatment outcomes derived from the ROC curves were: SUV_max-IN_ = 4.05 g/mL, MTV_IN_ = 1.85 cm^3^ and TLG_IN_ = 7.95 g. There were no significant associations between the residual metabolic burden below OC for all three metabolic parameters of the index nodes and oncological outcomes.

**Fig. 3:**
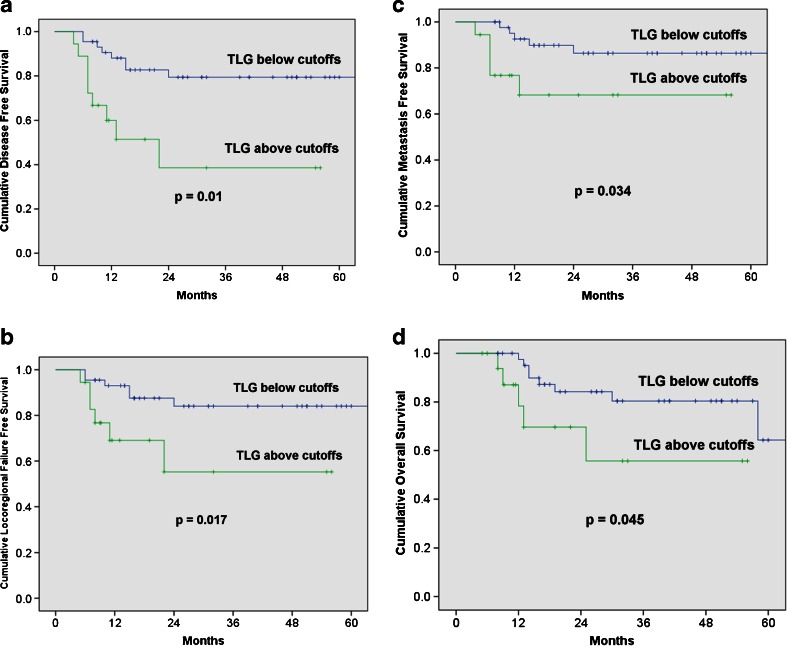
Oncological outcomes according to the total lesional glycolysis (TLG) of both primary tumour and index node during radiation therapy (RT) in patients with node positive disease. (**a**) Loco-regional failure-free survival; (**b**) Disease-free survival; (**c**) Metastasis-free survival; (**d**) Overall survival

In patients with node positive disease (*n* = 62), low metabolic values below OC (in both PT and IN) were associated with statistically significant better oncological outcomes. TLG was the best predictor of outcome with 2-year LRFS of 84.0 % vs. 55.3 % [*p* = 0.017, compared with SUV_max_ (*p* = 0.005) and MTV (*p* = 0.024)], DFS of 79.4 % vs. 38.6 % [*p* = 0.001, compared with SUV_max_ (*p* = 0.0004) and MTV (*p* = 0.002)], MFFS of 86.4% vs. 68.2 % [*p* = 0.034, compared with SUV_max_ (*p* = 0.073) and MTV (*p* = 0.042)] and OS of 80.4 % vs. 55.7 % [*p* = 0.045, compared with SUV_max_ (*p* = 0.077) and M TV(*p* = 0.049)]. Figure 3 shows the KM survival analysis results of TLG_PT+IN_ based on optimum cutoff values.

**Fig. 4:**
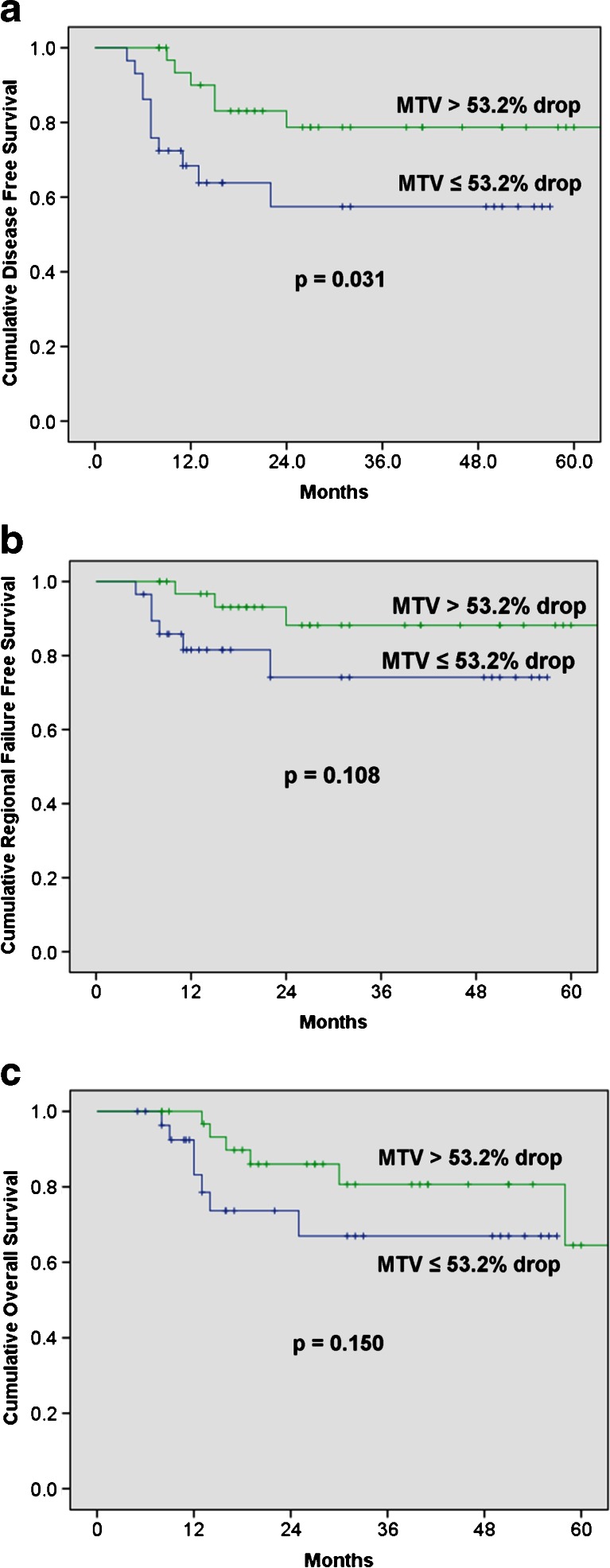
Oncological outcomes according to the percentage decrease in metabolic tumour volumes of the index node during radiation therapy (RT). (**a**) Disease-free survival; (**b**) Regional failure-free survival; (**c**) Overall survival

Optimal cutoffs of percentage reduction of IN for predicting treatment outcomes derived from the ROC curves were: SUV_max-IN_ = 45.3 %, MTV_IN_ = 53.2 % and TLG_IN_ = 67.1 %. As shown in Fig. 4, the percentage reduction in MTV_IN_ below the optimal cutoff (53.2 %) is the only parameter with significant association with DFS (*p* = 0.031) and a trend towards improved OS (*p* = 0.15).

## Discussion

To the best of our knowledge, this is the largest series for iPET and the first study to evaluate the role of TLG or MTV during RT in MPHNSCC. Our study is also the first study that assesses the prognostic value of mid-treatment nodal disease during RT. This study demonstrates that residual tumour metabolic burden of primary tumour, as measured by the FDG-PET metabolic parameters during the third week of primary RT for locally advanced MPHNSCC, can predict treatment outcome for loco-regional control and disease-free survival. Our findings are consistent with the hypothesis that the residual metabolic burden mid-treatment may correlate with tumour radiosensitivity. These iPET metabolic parameters could aid in the stratification of patients with poor or good treatment outcomes and allow for selection for adaptive therapy. In other words, it is likely to filter radioresistant disease, which may not be detected by pre-PET, at an early time point to allow individually adaptive radiotherapy.

There is limited data evaluating the role of FDG PET-CT in assessing early RT response at primary tumour site for MPHNSCC with mixed and inconclusive results, and most studies have used either visual analysis or reduction in SUV as criteria. Hentschel et al. performed three serial PET scans on 37 patients during RT for MPHNSCC (patients were divided between: after 10–20, 30–40 or 50–60 Gy), and found that an SUV_max_ decrease of ≥ 50 % was prognostic of loco-regional control and survival, but did not report the predictive accuracy of the test parameters [8]. Castaldi et al. evaluated the SUV changes based on modified EORTC criteria of 30 patients after 2 weeks of RT and failed to demonstrate any significant correlation with clinical outcomes [6]. Ceulemans et al. performed visual analysis of 40 patients after 47Gy and found that complete metabolic response had relatively low sensitivity and low positive predictive value for loco-regional control [5]. Chen et al. reviewed SUV_max_ and the reduction ratio of SUV_max_ (SRR) after cumulative dose of 40-50Gy during RT and found significant correlation of SRR with DFS and OS, but not between SUV_max_ and oncological outcomes [10]. In contrast to this, Farrag et al. reported that the SUV_max_ level after 4 weeks or 47 Gy was significantly associated with OS [11].

In our study, the third week of treatment was chosen pragmatically, as this was thought to be the most clinically relevant time frame in which a meaningful response may be assessed, but before significant inflammation occurs from RT and also allowing enough time for adaptation of treatment. In the third week, our results show TLG is a better and statistically more significant predictor of treatment outcome than SUV_max_ or MTV, consistent with our hypothesis that TLG can best reflect tumour metabolic burden, rather than relying on the highest intensity in a single voxel measured by SUV_max_. The prognostic value of TLG was more pronounced after the subgroup analysis (radiotherapy and chemotherapy only), since it was the only metabolic parameter showing significant associations with oncological outcomes (DFS and LRFS). Although larger studies with longer follow-up are required to validate our findings, it appears that TLG would be the most reliable prognostic indicator to assess the interim therapeutic response in MPHNSCC, especially when treated with radiation therapy and chemotherapy. Due to a small sample size (*n* = 15), further subgroup analyses were not performed for patients treated with Cetuximab concurrently with RT.

Consistent with some of published results, our study has shown that SUV_max_ reduction may not be ideal for early response monitoring in MPHNSCC. To match the methodology of published data [8], we assessed an SUV_max_ reduction of >50 % as a prognostic indicator, and found no significant difference in outcome. This finding may be explained by tumour heterogeneity, and the reduction in the metabolic burden probably represents killing of the more radiosensitive component of tumour, and the amount of residual metabolic burden is the more useful predictor of treatment outcome as it may give a measure of the volume of residual and/or radioresistant tumour. Another possible contributing factor is “rebound” FDG uptake due to treatment-induced inflammation. Inflammation related to RT can affect the measurements of FDG-PET. A study of the retention index using weekly dynamic PET on ten xenografts in mice with MPHNSCC undergoing 5 weeks of RT, reported that day 7/after 15Gy appeared to be the best time point in monitoring early response, taking into consideration the treatment-induced rebound FDG uptake [12]. This study also showed that retention index is superior to SUV_max_ in predicting treatment outcome.

We are aware of only one other clinical series assessing the prognostic value of residual metabolic burden. In that study, metabolic rate derived from plasma FDG level of iPET after 24 Gy of RT was found to be superior to SUV_max_ in predicting local control in MPHNSCC [7]. Routine calculation of MR in clinical practice is, however, not practical for most centres.

As shown in Table 4, all but two studies (our study and Hentschel et al.) included nasopharyngeal cancer, while our study was the only one that evaluated all metabolic parameters. The majority of studies used either IMRT and/or TomoTherapy®, excepting two (Hentschel et al. and Brun et al.). We excluded nasophayngeal cancers (NPC) since the majority of patients that attended our centres had the endemic type NPC, which followed a different natural history with a higher rate of distant recurrence compared to other MPHNSCC [13]. Unlike other published studies (Table 4), in order to avoid the heterogeneity which could affect the oncological outcome, we also excluded early stage patients (stage I and II) and patients treated with RT only.

**Table 4:** Studies evaluating the predictive role of metabolic parameters of 2-[18F] fluoro-2-deoxy-D-glucose positron emission tomography-computed tomography performed during radiation therapy in head and neck cancer

Studies	Number	Tumour subsites	Overall stage	Primary treatment	Median Follow up (months)	When	PET parameters	Outcome endpoints	Significance
Our study	72	OCC, OPC, LRC, HPC	IIIx18, IVx54	CRT×57, Cet-RT×15	25 (6–70)	3rd week	SUV_max_, MTV, TLG	DFS, LRFS, MFFS, OS	Yes (SUVmax, MTV, TLG with DFS, LRFS)
Chen et al. 2014 [10]	51	OPC, HPC, NPC	IIIx16, IVx35	RT×7, CRT×41, Cet-RT × 3	23 (7–53)	Cumulative dose of 40-50Gy	SUV_max_, SRR	DFS, PRFS, NRFS, OS	Yes (SRR-P with DFS and OS)
Castaldi et al., 2012 [6]	26	OPC, HPC, NPC, LRC	IIx1, IIIx7, IVx18	CRT	29.2 (2.8-56)	After 2 wks	SUV_max_	RFS and DFS	No
Hentschel et al., 2011 [8]	37	OCC, OPC, HPC, LRC	Not clear (only T and N stage reported)	CRT	26 (8–50)	10-20 Gy/week 1or2) 14 to 21 days (range: 1st to 6th week)	SUV_max_, GTV PET	LRFS, DFS, OS	Yes (SUV_max_ >50 % reduction after 10-20Gy or week 1-2 with OS)
Ceulemans et al. 2011 [5]	40	OCC, OPC, NPC, HPC, LRC	Ix2, IIx9, IIIx10, IVx19	RT×34, CRT×16	26 (7–50)	Week 4/ 47Gy	CR/NCR (visual assessment)	OS	No (CR with OS)
Farrag et al. 2010 [11]	43	NPC, LRC, HPC, OPC, OCC	Not clear (only T and N stage reported)	RT×27, CRT×16	Median 12.7 months (3–34.5)	After 4 weeks or 47Gy	SUV_max_	DFS, OS	Yes (SUV_max_ with OS)
Brun et al. 2002 [7]	47	OCC, OPC, HPC, LRC, OTHERS	II-IIIx17, IVx30	RT × 37, IC+CRT×10, RT+Sg×1, RT+Sg+BR×1	39.6 (14.4-82.8)	1-3 weeks	MR + SUV	CR (Complete Remission, LC, OS	Yes (MR with CR, LC, OS).

OCC = oral cavity cancer; OPC = oropharyngeal cancer; LRC = laryngeal cancer; HPC = hypopharyngeal cancer; NPC = nasopharyngeal cancer; RT = radiation therapy; CRT = concurrent chemotherapy and RT; Cet-RT = Cetuximab+RT; SUV_max_ = maximum standardised uptake value; MTV = metabolic tumour volume; TLG = total lesional glycolysis; DFS = disease-free survival; LRFS = loco-regional failure-free survival; MFFS=metastatic failure free survival; OS = overall survival; CR = complete response; NCR = non-CR; RFS = relapse-free survival; LC = local control; MR = metabolic rate; Gy = gray; Sg = surgery; BR=brachytherapy; GTV = gross tumour volume; PRFS = primary RFS; NRFS = nodal RFS; SRR = Reduction Ratio of SUV_max_

There is no published literature on the prognostic value of mid-treatment nodal response in patients with MPHNSCC undergoing primary RT. In head and neck cancer, the largest node with highest metabolic burden (measured by TLG and defined as “index node” in our series) is likely to be the most predictive of treatment outcome, and most reproducible across different centres. Therefore, we decided to assess the metabolic parameters of IN and its correlation with treatment outcomes. In our study, the response rate rather than the residual metabolic burden of IN appears to predict the tumour outcome in nodal disease. The percentage reduction of MTV was found to be the only prognostic indicator of DFS. This suggests that the therapeutic response of the nodal disease may not be the same as the primary tumour. On the other hand, the predictive value of residual metabolic burden measured by all three metabolic parameters was improved significantly when both PT and IN were combined. To our knowledge, there is no study that reports a functional imaging study that is predictive of distant failure in non-nasopharyngeal MPHNSCC. In our study, in patients with nodal disease, TGL_PT+IN_ is predictive of the distant failure rates and overall survival. This information is likely to be useful, especially in adaptive systemic therapy trials, and therefore, should be validated in larger prospective studies.

One limitation of this study is that tumour grading and HPV status are not available for the majority of patients, but we believe that correlation with HPV status may better identify patients suitable for dose de-intensification. We are currently evaluating the feasibility of deriving this information retrospectively, and correlating this with the prognostic significance of pre-PET and iPET metabolic parameters. Further biological profiling in iPET such as tumour hypoxia or proliferation indices may better explain the radiobiology for possible tumour radioresistance in patients with high residual metabolic burden, and identify the best strategy for adaptive therapy. In addition, assessment of nodal metabolic status in iPET may also provide additional prognostic information and improve correlation with treatment outcomes, particularly OS.

Although we have used week 3 for iPET as a clinical time point to allow enough cell kill and time for adapting therapy, the optimal time to perform iPET remains undecided. Despite this, we have demonstrated the utility of week 3 iPET to identify patients with poor and good treatment outcome for selection for possible adaptive therapy. Future studies with serial dynamic PET assessing all metabolic parameters may be of value to further improve the predictive sensitivity and specificity with iPET. Localisation of high risk areas should also be evaluated, including determining the role of functional magnetic resonance imaging to target radioresistant subvolumes within the gross tumour.

## Conclusion

The metabolic parameters of PT and IN of iPET can be useful predictors of patient outcome and have a potential role in adaptive therapy for MPHNSCC. Among the three parameters, TLG was found to be the best prognostic indicator of oncological outcomes.
